# Effects of disulfide bridges and backbone connectivity on water sorption by protein matrices

**DOI:** 10.1038/s41598-017-08561-2

**Published:** 2017-08-11

**Authors:** Sang Beom Kim, Rakesh S. Singh, Prem K. C. Paul, Pablo G. Debenedetti

**Affiliations:** 1 Department of Chemical and Biological Engineering, Princeton University, Princeton, New Jersey, 08544 United States; 2 Unilever R&D, Port Sunlight Laboratory, Wirral, CH63 3JW United Kingdom

## Abstract

Understanding the water sorption behavior of protein powders is important in applications such as the preservation of protein-based pharmaceuticals. Most globular proteins exhibit a characteristic sigmoidal water adsorption isotherm at ambient conditions. However, it is not well understood how water sorption behavior is influenced by intrinsic factors that are related to structural properties of proteins. We investigate computationally how structural constraints on proteins influence the water sorption isotherms of amorphous protein powders. Specifically, we study the effects of non-local disulfide linkages and backbone connectivity using pheromone ER-23 and lysozyme as model proteins. We find that non-local disulfide linkages can significantly restrict structural changes during hydration and dehydration, and this in turn greatly reduces the extent of hysteresis between the adsorption and desorption branches. Upon removing the backbone connectivity by breaking all peptide bonds in lysozyme, we find that the hysteresis shifts towards the lower humidity regime, and the water uptake capacity is significantly enhanced. We attribute these changes to the higher aggregation propensity of the constraint-free amino acids in dehydrated condition, and the formation of a spanning water network at high hydration levels.

## Introduction

Acquiring fundamental understanding of protein-water interactions is important in numerous practical applications involving protein-based products, such as pharmaceuticals and biomaterials^1–6^. For example, enzymes such as lysozyme can only function properly in the presence of a threshold amount of water^3^. Knowledge of the water sorption behavior of human and animal hair, which is largely composed of proteins, influences the development of cosmetics and textile products^7^. In addition, understanding protein-water interactions at low-moisture conditions is important in the pharmaceutical industry, where it could help in the formulation of effective strategies to mitigate any irreversible damage to proteins during the lyophilization process^5, 6, 8^. It is therefore important to obtain molecular-level insights on protein-water interactions and the factors that could modulate these interactions, especially at small water contents. One way of studying protein-water interactions systematically for a range of hydration levels of practical interest is through water sorption experiments^1^. The goal of such experiments is to understand the equilibrium relationship between the moisture content of a protein powder or protein-based matrix and the relative humidity of the surrounding vapor at a given temperature and pressure. This is known as the powder’s sorption isotherm.

The water sorption isotherms of a variety of protein-based products have been measured across a range of conditions, in order to understand the effects of process factors that could potentially affect protein-water interactions^4–6, 9–11^. Excipients such as salts and biomolecules are key factors that affect protein-water interactions on account of competitive binding between water and excipient molecules to proteins^12–14^. At the isoelectric pH the net charge of the protein molecule is zero, and the resulting protein structures are generally compact, which in turn minimizes protein-water interactions^12^. Changes in pH alter protein charges, and can therefore affect water sorption behavior^12^.

Apart from these external factors, there are also intrinsic factors (characteristic of the protein itself) such as the number (chain length) and composition (sequence) of the amino acids, secondary/tertiary structures, backbone connectivity, and structural flexibility of the protein matrices, that could potentially affect the water sorption behavior by modulating the nature of protein-water interactions. These intrinsic factors could, for example, influence the accessibility of hydrophilic residues to the water molecules, thereby altering protein-water interactions^12–18^. Recently, it has been shown computationally that water sorption isotherms are significantly affected when a protein’s flexibility^19, 20^ and its fraction of charged amino acids^21^ are altered. However, despite numerous water sorption studies, and the well-known fact that most globular proteins and protein products at ambient conditions exhibit a generic sigmoidal water sorption isotherm (type II in IUPAC convention^22^) with a pronounced hysteresis between adsorption and desorption branches, the effects of the above-mentioned intrinsic factors on water sorption behavior have not been studied systematically, and hence remain poorly understood.

A major challenge in gaining fundamental understanding of water sorption isotherms lies in the lack of appropriate spatio-temporal resolution in experiments, and, until recently, in the comparative lack of computational methods that are adequate for simulating the isotherms^19, 23^. Previously, hybrid techniques that combine molecular dynamics (MD) and grand canonical Monte Carlo (GCMC) have been used to simulate sorption isotherms of flexible adsorbents, but it has been challenging to ensure proper homogenization of MD and GCMC, which is essential for microscopic reversibility, and to attain satisfactory computational efficiency^19, 24, 25^. Recent advances in simulation methods^23^, however, have enabled the efficient simulation of water sorption isotherms of flexible protein matrices using an exclusively MD-based approach.

In this work, we employ advanced computer simulation techniques to investigate the effects of structural constraints on the water sorption isotherms of amorphous protein matrices. In particular, we control disulfide linkages and backbone connectivity, both of which are key to protein structure and functionality. We performed simulations on amorphous powders instead of crystals or single-protein systems because water sorption experiments are commonly done on lyophilized protein powders^3^. Disulfide bridges are formed between a pair of thiol groups (–C–SH) in a protein’s cysteine residues. Understanding the effects of disulfide bridges is of direct relevance to the water sorption behavior of hair, which contains a significant amount of proteins with high sulfur content^26^. Amino acids are connected linearly through peptide bonds to form a protein chain, and this imposes structural constraints that result in the highly specific three-dimensional structures of proteins. We show how the disulfide linkages and backbone connectivity affect the water sorption behavior of protein matrices by influencing the structural changes experienced by the protein during the adsorption and desorption processes.

## Results

### Effects of non-local disulfide linkages on the water sorption behavior of pheromone ER-23

**Figure 1:**
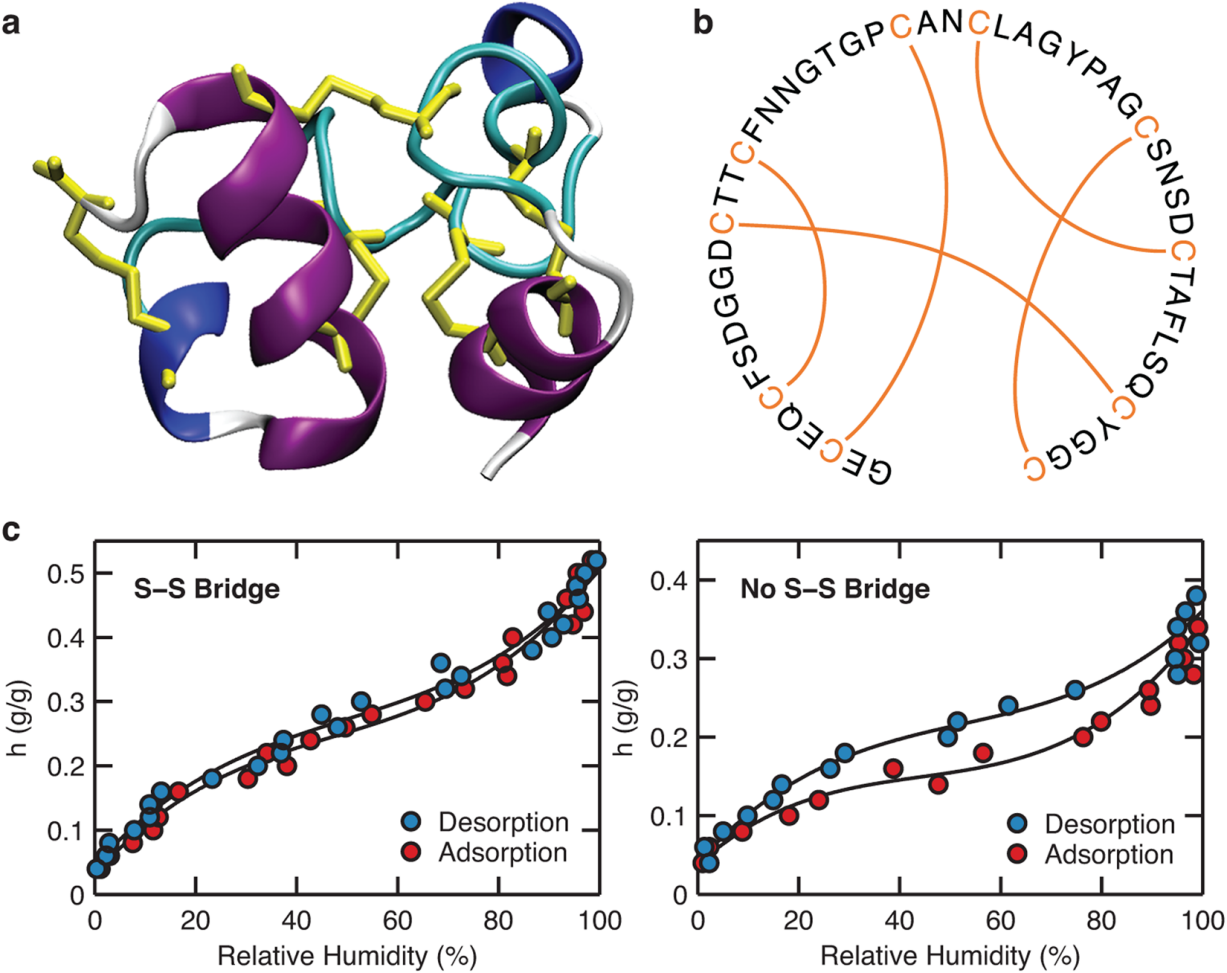
Visualizations of the (**a**) three-dimensional structure and (**b**) sequence of pheromone ER-23. There are five disulfide bridges, shown explicitly by yellow bonds (**a**) and orange lines (**b**). (**c**) Water sorption isotherms of pheromone ER-23 with (left) and without (right) disulfide bridges. Black lines are drawn as a guide to the eye.

We chose pheromone ER-23^27^, a cysteine-rich signaling protein from the ciliated protozoan *Euplotes raikovi*, as our model system to study the effects of disulfide linkages on water sorption behavior. It has a well-characterized structure with a similar cysteine content as the high-sulfur proteins in hair (approximately 20% in number of residues)^26^; the latter, however, are challenging to study computationally due to uncertainties in structural characterization. The structure and sequence of pheromone ER-23 are shown in Fig. 1a,b, respectively. The five disulfide bridges between the sulfur atoms in cysteine residues are explicitly shown in each case. The illustrations show that the connections formed by the disulfide bridges are non-local, but instead closely connect sequentially distant residues. We computed the water sorption isotherms for pheromone ER-23 at 300 K and 1 bar, with and without disulfide bridges (Fig. 1c). When the disulfide bridges are not present, the isotherm exhibits the typical Type-II shape with a pronounced hysteresis between the adsorption and desorption curves. The isotherm saturates at a hydration level (*h*) of approximately 0.3 g water/g dry protein (g/g), above which the hydration water starts exhibiting bulk-like characteristics. With the disulfide bridges, however, we observe that the magnitude of hysteresis is significantly reduced.

**Figure 2:**
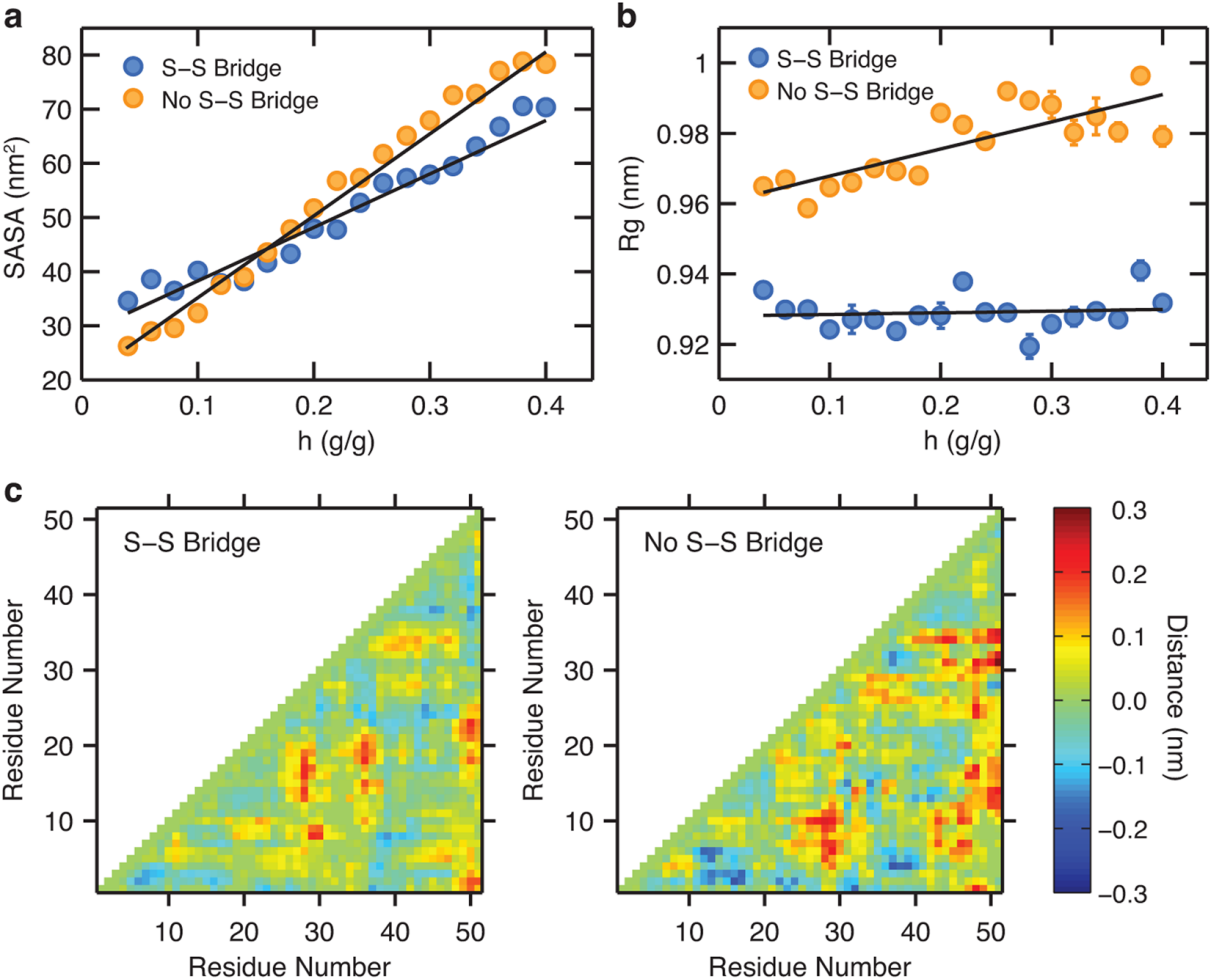
(**a**) Solvent-accessible surface area (SASA) and (**b**) radius of gyration (Rg) as a function of hydration level (*h*) for the adsorption process. To illustrate the differences in the dependence of SASA and Rg on *h*, the best-fit linear lines are shown in black. The error bars are either explicitly shown or smaller than the size of the symbols. (**c**) Contact maps that show the distances between residue pairs at *h* = 0.4 g/g after subtraction from the corresponding ones at *h* = 0.04 g/g.

We attribute this difference in the water sorption behavior to the increased rigidity of the protein in the presence of disulfide linkages. To study the effects of disulfide linkages on hydration-dependent structural changes, we first show the solvent-accessible surface area (SASA) of the entire powder as a function of hydration level (Fig. 2a). As the hydration level increases, the SASA increases as well due to the increased exposure of the hydrophilic residues. In the presence of disulfide bridges, pheromone ER-23 exhibits a slightly larger SASA when dehydrated, but the rate of increase in SASA with hydration level is smaller, eventually having less SASA at sufficiently high levels of hydration (*h* > 0.15 g/g) when compared to pheromone ER-23 with no disulfide bridges. A similar trend is observed when the radius of gyration (Rg) is analyzed (Fig. 2b), which we computed by averaging the Rg of individual proteins in the powder. While the Rg of pheromone ER-23 increases with hydration level when no disulfide bridge is present, it is relatively constant in the presence of disulfide bridges.

In order to investigate local, residue-level structural changes, we also computed distance maps at two hydration levels, 0.04 and 0.4 g/g, where the distance between each pair of residues within a protein is represented using a color map (see also Supplementary Fig. S1). The inter-residue distance was measured by taking the shortest distance between any pair of atoms from the two residues. This was computed for each configuration in the simulation trajectory and then averaged. When protein powders are hydrated, the inter-residue distance generally increases due to the swelling of the protein matrices. Figure 2c shows the change in the distances between residue pairs when each powder system was hydrated from 0.04 g/g to 0.4 g/g. When the disulfide bridges are not present, there is a significantly larger overall inter-residue separation due to the hydration, which is consistent with the hydration-level dependence of Rg (Fig. 2b).

The SASA, Rg, and inter-residue distance results indicate that the disulfide bridges effectively increase the rigidity of the proteins by restraining their conformational changes. Palmer *et al*. previously showed, using ubiquitin as a model system, that hysteresis disappears when this protein is treated as perfectly rigid at its fully hydrated structure^19^. That study, along with the present observation of reduction of hysteresis in the presence of disulfide linkages, suggests a close connection between rigidity of proteins and shrinkage of hysteresis between the adsorption and desorption isotherms. Recently, we identified residue-level differences in the local hydration of charged/polar residues between the adsorption and desorption processes as the microscopic signature of hysteresis in water sorption isotherms^21^. Following the same procedures^21^, we characterized the difference in local, residue-level hydration between adsorption and desorption processes at a hydration level of 0.2 g/g, where the magnitude of hysteresis is the largest (maximum difference in relative humidity between adsorption and desorption branches) in the absence of disulfide bridges (see Supplementary Fig. S2). We find that in absence of disulfide bridges pheromone ER-23 clearly exhibits residue-level differences in hydration between adsorption and desorption, which predominantly occur at charged or polar residues (e.g. 12D, 18N, 24C, and 39D). In contrast, the magnitude of local hydration differences diminishes significantly when disulfide bridges are present. In case of a flexible protein matrix, proteins undergo local conformational changes during hydration/dehydration, which result in residue-level differences in solvent accessibility and local hydration behaviors between adsorption and desorption processes. Ideally rigid proteins, however, exhibit no such differences in local hydration due to the lack of structural changes, which leads to a suppression of hysteresis. The significantly reduced hysteresis of pheromone ER-23 powder in the presence of disulfide bridges can therefore be attributed to the increase in rigidity caused by linkages between non-vicinal cysteine residues. The extent of increase in rigidity by the disulfide bridges would greatly depend on the fraction of disulfide bridge-forming cysteine residues in the protein.

### Effects of backbone connectivity on the water sorption behavior of lysozyme

**Figure 3:**
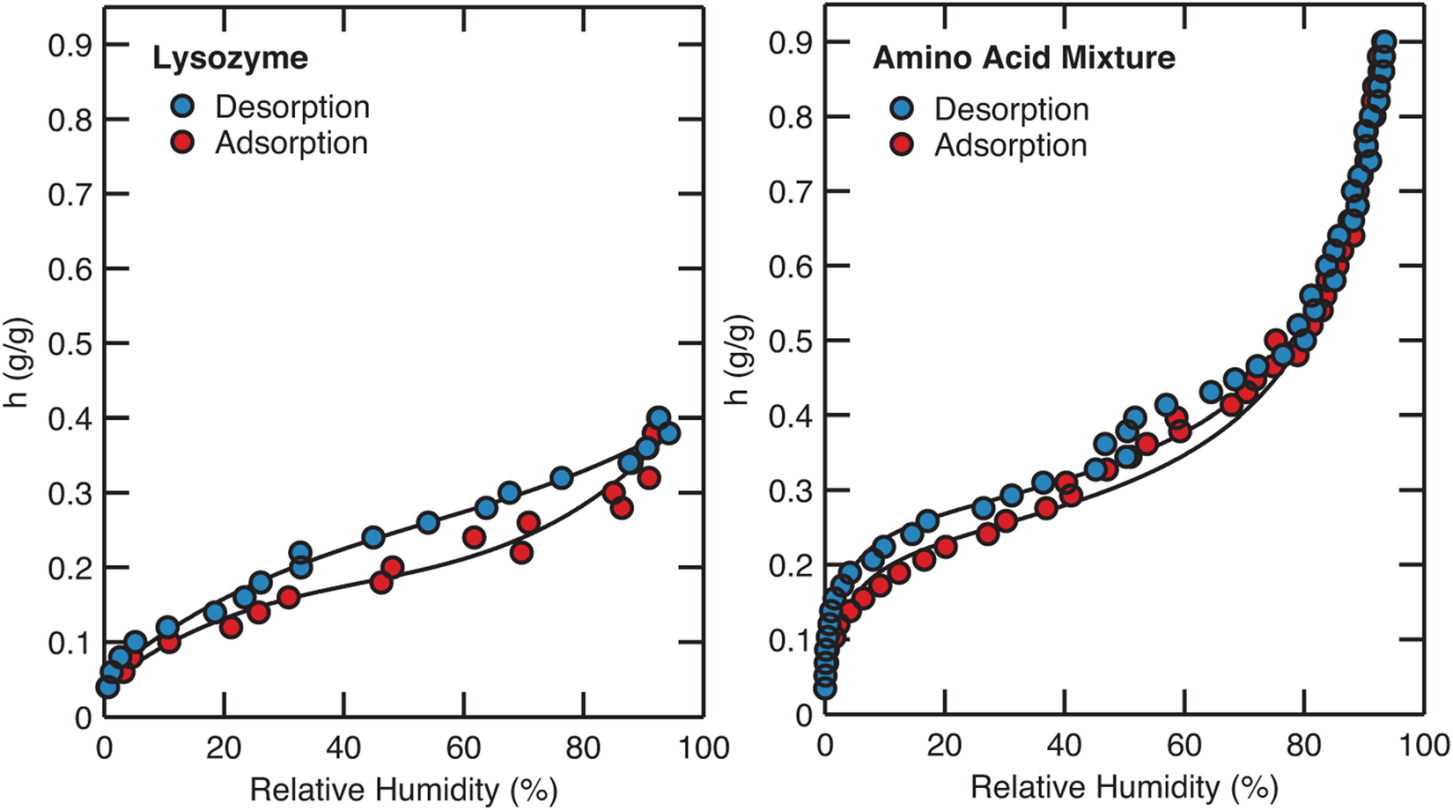
Water sorption isotherms of lysozyme (left, reprinted with permission from Kim *et al*. *J*. *Phys*. *Chem*. *Lett*., 8, 1185–1190 (2017)^21^. Copyright 2017 American Chemical Society.) and the amino acid mixture of the same composition as lysozyme (right). Black lines are drawn as a guide to the eye.

We now explore the effect of backbone connectivity on water sorption behavior. For this study we chose lysozyme and the corresponding free amino acid mixture (same amino acid composition as lysozyme) as our model systems. Lysozyme is widely used as a model protein in many studies due to its functional importance in antibacterial resistance and its availability at low cost^28, 29^. Figure 3 shows the comparison between the water sorption isotherms of lysozyme and the corresponding amino acid mixture. The lysozyme isotherm shows the Type-II shape that is typically observed in globular proteins, with saturation at a hydration level of approximately 0.4 g/g. In contrast, the isotherm of the corresponding amino acid mixture exhibits a significantly reduced hysteresis with a saturation point above a hydration level of approximately 0.9 g/g. This shows that the lack of backbone connectivity results in greater water uptake at a given relative humidity and less pronounced hysteresis between adsorption and desorption processes. In addition, the hysteresis is mainly present in the comparatively low humidity regime (below approximately 30%) with a smaller amplitude compared to that in the lysozyme isotherm, and it disappears at higher hydration levels.

**Figure 4:**
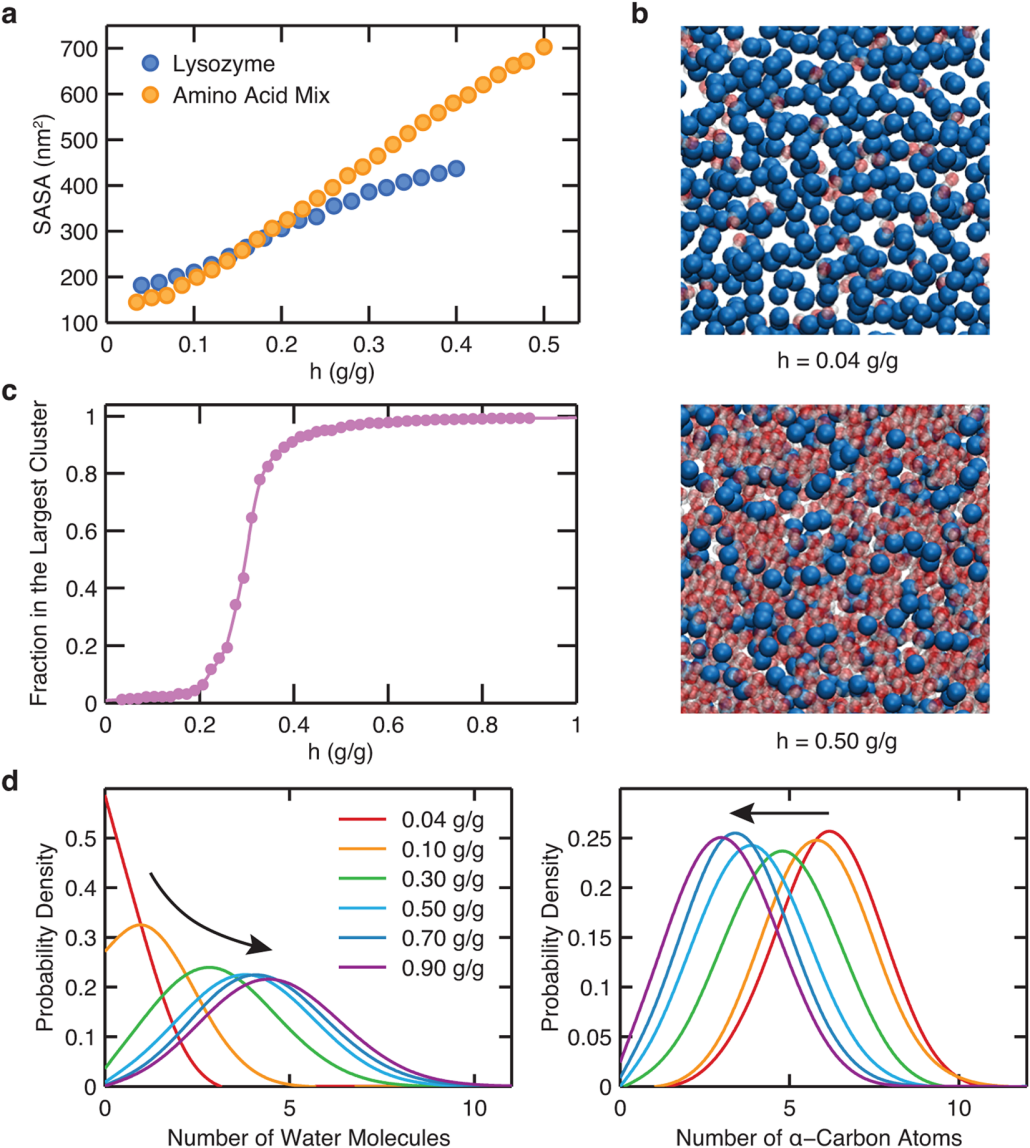
(**a**) Solvent-accessible surface area (SASA) as a function of hydration level during the adsorption process. The error bars are either explicitly shown or smaller than the size of the symbols. (**b**) Visualization of the *α*-carbons (blue) and the water molecules (red) of the amino acid mixture at low (*h* = 0.04 g/g, top) and high (*h* = 0.50 g/g, bottom) hydration levels. (**c**) Fraction of water molecules that belong to the largest cluster as a function of hydration level. Best-fit line is drawn as a guide to the eye. (**d**) Probability distribution of the number of water molecules (left) and *α*-carbon atoms (right) within the first coordination shell from the *α*-carbon atoms of the amino acid mixture at varying hydration levels. Although these distributions are discrete in nature, we show them as continuous for visual clarity in comparing among different hydration levels. Arrows indicate the direction of increase in hydration level.

In order to understand the underlying structural difference between lysozyme and its corresponding amino acid mixture, we computed the SASA for each system as a function of hydration level (Fig. 4a). At low hydration levels (*h* < 0.15 g/g), lysozyme has a slightly larger SASA than the amino acid mixture. This is because the free amino acids have a high propensity to interact among themselves and aggregate when dehydrated, whereas the constraints imposed by lysozyme’s backbone connectivity effectively hinder this aggregation propensity. At higher levels of hydration (*h* > 0.15 g/g), on the other hand, the SASA of the amino acid mixture is larger than that of lysozyme, and it increases monotonically with hydration level unlike the lysozyme SASA, which exhibits signatures of saturation near a hydration level of 0.4 g/g. Upon hydration, more water molecules become available to solvate and interact with amino acids; thus, the amino acids tend to de-aggregate and expose more surface area to the solvent. However, unlike the amino acid mixture, the residues in lysozyme are restricted by backbone connectivity and thus cannot comparably separate from each other upon hydration.

In Fig. 4b, we show snapshots of the amino acid mixture at two hydration levels, 0.04 and 0.5 g/g. At the low hydration level, the amino acids show a propensity to interact among themselves due to the insufficient number of dispersed water molecules. In order to obtain a deeper quantitative insight into water distribution within the amino acid mixture, we performed a clustering analysis of water molecules, where we define a cluster as a network of water molecules that are connected with each other. Two water molecules are considered to be connected if their oxygen-to-oxygen distance is less than 3.5 Å, which is the cut-off distance commonly used to define the first hydration shell in bulk water^30^. In Fig. 4c, we show the fraction of water molecules that belong to the largest water cluster in the amino acid mixture matrix. It can be seen that this quantity exhibits a sigmoidal dependence on the hydration level. As is also evident from the simulation snapshots in Fig. 4b, at low hydration levels (*h* < 0.2 g/g) most of the water molecules do not belong to a common cluster, and they are distributed in small pockets, selectively solvating few amino acids. By contrast, upon increasing the hydration level to above approximately 0.4 g/g, water molecules form a spanning network embedding the amino acid mixture matrix.

To probe the local hydration of the amino acid mixture, we show in Fig. 4d the distributions of the number of water molecules and *α*-carbons in the first hydration and coordination shells, respectively, of *α*-carbons. A water molecule is considered to be in the first hydration shell of an *α*-carbon if the oxygen atom in the water molecule is within 4.3 Å from the *α*-carbon, with this threshold distance determined by the first minimum in the radial distribution function of *α*-carbon and water oxygen atoms (see Supplementary Fig. S3). Similarly, the first coordination shell of *α*-carbons is defined using an *α*-carbon–*α*-carbon distance of 6.3 Å, based on the *α*-carbon–*α*-carbon radial distribution function (see Supplementary Fig. S3). Upon increasing the hydration level, the average number of water molecules in the first hydration shell of *α*-carbons increases, whereas the average number of *α*-carbons in the first coordination shell of *α*-carbons decreases. Our calculations therefore show that the increase in water content leads to enhanced solvation in the vicinity of the amino acids, leading eventually to a spanning network of water molecules embedding the matrix, with a concomitant increase in the separation between amino acids.

We therefore obtain the following physical picture of the hydration-dependent structural change of the free amino acid mixture. At low hydration levels, the free amino acids tend to aggregate, exhibiting similar behavior to lysozyme, where hydrophilic residues are preferentially exposed to water. However, at higher levels of hydration, the free amino acids separate from each other without the constraints arising from backbone connectivity, and this leads to enhanced water adsorption capability and SASA relative to the lysozyme powder.

As mentioned earlier, we have recently identified a correlation between the water sorption hysteresis and localized, residue-level differences in hydration that occur between the adsorption and desorption branches, even at the same water content^21^. Such local differences occur mostly at charged or polar residues, which are often exposed to water while the non-polar residues are buried in the protein’s interior core. The lysozyme powder exhibits hysteresis across a wide span of relative humidity, because lysozyme maintains a relatively compact globular structure, even at high humidity levels. Water molecules hydrate the exposed polar/charged residues of lysozyme differently between adsorption and desorption processes. The amino acid mixture shows hysteresis at low humidity, reflecting history-dependent access by water molecules to the aggregating, poorly hydrated mixture. At high hydration levels, however, the amino acid mixture exhibits a significant reduction in hysteresis due to the formation of a spanning water network, which embeds the amino acids and greatly diminishes localized (preferential) amino acid hydration.

## Discussion

In this work, we have shown that the water sorption behavior of amorphous protein matrices is greatly influenced by structural constraints, which are studied here by modulating non-local disulfide bridges and backbone connectivity in pheromone ER-23 and lysozyme, respectively. The non-local connectivities made by disulfide bridges in pheromone ER-23 effectively increase the protein’s rigidity by restricting structural changes. Using SASA, Rg, and pairwise distances between residues as metrics, we showed that pheromone ER-23 exhibits limited structural changes during the sorption process. Accordingly, the water sorption isotherm of pheromone ER-23 with disulfide bridges exhibits only minor hysteresis between adsorption and desorption. This is consistent with past findings on ubiquitin, where Palmer *et al*. observed disappearance of hysteresis when the structure of ubiquitin was completely constrained during the water sorption simulations^19^.

The backbone connectivity also significantly affects the water sorption isotherms. Using lysozyme as our model system, we explicitly compared the isotherms of lysozyme and the corresponding mixture of amino acids with the same composition as lysozyme. The lack of backbone connectivity significantly reduces the hysteresis and increases the water adsorption capacity of the system. Additionally, we also observe that the hysteresis between adsorption and desorption isotherms is suppressed at high relative humidity. We attribute these observations to the higher propensity for separation among the amino acids at high levels of hydration, along with the formation of network of spanning water molecules that embeds the amino acids. In contrast, lysozyme has a comparatively limited capacity for accommodating changes in inter-residue separations due to the constraints imposed by backbone connectivity. This adsorption behavior of the amino acid mixture at lower hydrations has some resemblance with lysozyme in the sense that in both cases not all amino acids are equally exposed to water. This is consistent with recent observation that hysteresis correlates with localized differences in hydration^21^.

Potentially interesting future avenues of inquiry include investigation of water sorption isotherms of a more heterogeneous system. Protein powders typically contain excipients such as carbohydrates or salts in order to protect the proteins from denaturation and to control the pH of the system. Characterizing the effects of the cosolutes would provide important insights on the water sorption behavior of industrially-realistic protein powders. In previous work^23^ we have shown that powders made of folded and unfolded Trp-cage monomers exhibit indistinguishable adsorption isotherms. It would be interesting to test whether this is a general result, and in particular whether it applies to lysozyme.

## Methods

### Water Sorption Isotherm Calculation

The protein structures for the water sorption isotherm simulations were taken from RCSB Protein Data Bank (PDB ID’s: 1HA8 (pheromone ER-23)^27^ and 1AKI (lysozyme)^31^). The amorphous protein powder systems were created by first placing multiple proteins (4 for pheromone ER-23 and 8 for lysozyme) in a simulation box with random translation and rotation. The system was then solvated with water molecules (8,700 and 24,500 water molecules for pheromone ER-23 and lysozyme systems, respectively) and equilibrated for 500 ns with NPT MD simulation at 300 K and 1 bar. The simulations of adsorption and desorption processes were performed by following the methods of Kim *et al*.^23^.

Each protein powder was first dehydrated to *h* = 0.04 g/g, which is approximately the amount of residual water in freeze-dried protein powders^6^. This initial dehydration was performed through cycles comprising of removal of one water molecule and relaxation of the system for 200 ps using NPT MD at 300 K and 1 bar. We randomly chose the water molecule to be removed based on the weight of the Boltzmann factor (*e* ^−*β*Δ*U*^), where *β* is the inverse of the product of temperature and Boltzmann’s constant, and Δ*U* is the change in configurational energy (*U*) that would be caused by removing the specific water molecule. Each system was then hydrated up to the approximate saturation limit (0.5 g/g for pheromone ER-23 with disulfide bridges, 0.4 g/g for pheromone ER-23 without disulfide bridges, 0.4 g/g for lysozyme, and 0.9 g/g for the amino acid mixture) for adsorption simulation, and then dehydrated back to 0.04 g/g for desorption.

The adsorption simulation was performed by inserting each water molecule in a “continuous” rather than “discrete” manner^23^. Each inserted water molecule has a gradually increasing interaction from ideal-gas limit to a full interaction. The configurational energy of the system can be described using the following equation:

1 $$U(\lambda )={U}_{rest}+[\lambda (t)\times {U}_{inserted}(\lambda )],$$

where *λ* is the coupling parameter that ranges from 0 (no interaction) to 1 (full interaction). First, a water molecule is inserted at a non-overlapping random position in the simulation box, with no interaction with the rest of the system (*λ* = 0). Then its interaction is gradually increased until the full interaction limit is reached (*λ* = 1) while performing a 400 ps of NPT MD simulation. The insertion is repeated until a hydration level of interest is reached, and the dehydration (desorption) process is executed the same way except that the interaction of a randomly selected water molecule is gradually turned off.

Configurations were saved every *h* = 0.02 g/g during the sorption processes for the 700 ns relaxation through NPT MD simulation (300 K and 1 bar), of which the last 600 ns was used for the data analysis. Relative humidity is defined as $100\times \exp \,(({\mu }_{w}-{\mu }_{w}^{sat})/(RT))$, where *μ*_*w*_ is chemical potential of water, ${\mu }_{w}^{sat}$ is chemical potential of saturated water vapor, *T* is absolute temperature, and *R* is the gas constant. Bennett’s acceptance ratio method^32^ was used to compute *μ*_*w*_, which performs numerous insertions and deletions of test water molecules. Consistent with our previous work^21^, for each simulation frame we performed 20 × 10^6^ insertions and a number of deletions that corresponds to the number of water molecules in the system. Chemical potential of saturated water vapor (${\mu }_{w}^{sat}$) was calculated using the ideal gas law and the vapor pressure of the SPC/E water model at 300 K (0.01 bar)^33^.

### Molecular Dynamics Simulation

The GROMACS^34, 35^ simulation package was used for all MD simulations. Equations of motion were integrated using the leap-frog algorithm with a time step of 2 fs. The temperature and pressure of 300 K and 1 bar, respectively, were controlled using Nosé-Hoover thermostat^36, 37^ with a 0.2 ps time constant and the Parrinello-Rahman barostat^38, 39^ with a 2 ps time constant. The independent fluctuations in the three dimensions of the simulation box were imposed through anisotropic pressure coupling. Periodic boundary conditions were applied in all three dimensions. We truncated the short range interactions at 1 nm, and the standard long-range dispersion corrections were made for the pressure and energy^40^. We used the smooth-particle mesh Ewald method^41^ to compute the reciprocal part of the Ewald sum of the long-range electrostatics. The linear constraint solver algorithm (LINCS)^42, 43^ and SETTLE^44^ were used to constrain all bonds in the protein and water molecules, respectively. CHARMM27^45^ protein force field was used in combination with SPC/E^46^ water model, with the Lorentz-Berthelot combining rules. The accuracy of this combination of force fields in simulating water sorption isotherms has been validated in a previous study^21^. Images of the protein structures were rendered using Visual Molecular Dynamics (VMD)^47^. The standard errors were computed using the block-averaging analysis^48^, by dividing each simulation trajectory into 5 equal-sized blocks (120 ns).

## Electronic supplementary material

Supplementary information
