# Supplementary material for: Effects of disulfide bridges and backbone connectivity on water sorption by protein matrices
Source: Sci Rep. 2017 Aug 11;7:7957. doi: 10.1038/s41598-017-08561-2 (PMC5554179; doi:10.1038/s41598-017-08561-2)
Supplement: Supplementary file 1 — Supplementary information [file 41598_2017_8561_MOESM1_ESM.pdf]

Supplementary Information

Effects of disulfide bridges and backbone connectivity on water sorption by protein matrices

Sang Beom Kim[1], Rakesh S. Singh[1], Prem K. C. Paul[2], and Pablo G. Debenedetti[1,*]

[1]*Department of Chemical and Biological Engineering, Princeton University, Princeton, New Jersey 08544, United States*

[2]*Unilever R&D, Port Sunlight Laboratory, Wirral, CH63 3JW, United Kingdom*

*Corresponding author: pdebene@princeton.edu

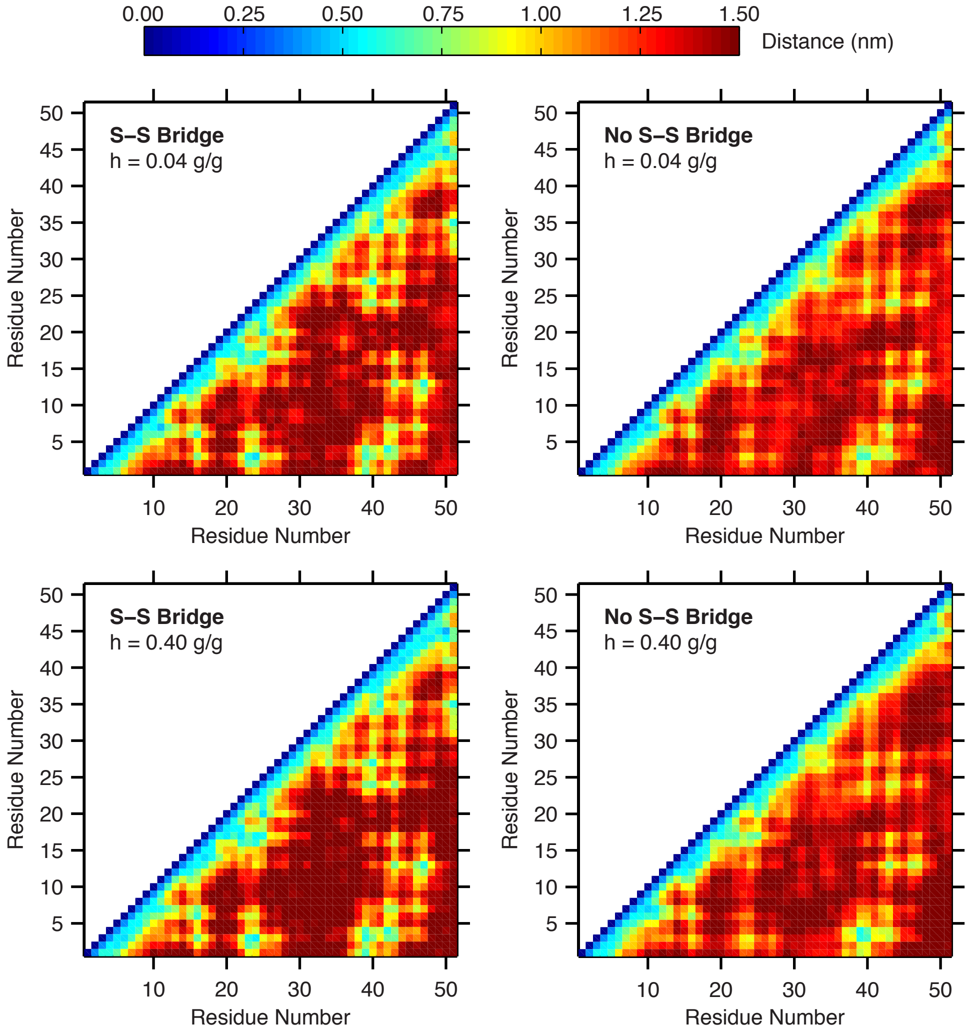

Figure S1. Contact map for pheromone ER-23 with (left) and without (right) disulfide bridges, calculated at two hydration levels (0.04 g/g – top and 0.40 g/g – bottom).

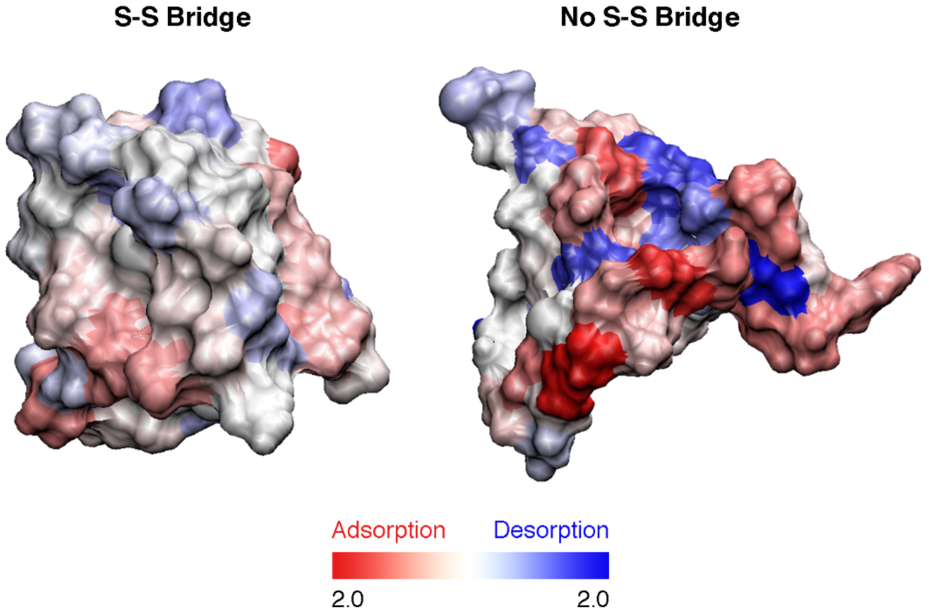

Figure S2. Visualization of differences in water density for each residue (number of water oxygen atoms within 0.31 nm from any non-hydrogen residue atom) in pheromone ER-23 in the presence (left) and absence (right) of disulfide bridges. A residue is shown in red if it has a greater local water density during adsorption, and blue if it is more locally hydrated during desorption. Residues with white colors show no difference between the adsorption and desorption processes. The calculation was done at hydration level of 0.2 g/g, at which the pheromone ER-23 powder without disulfide bridges exhibit the maximum degree of hysteresis. The 0.31 nm cut-off distance was determined from the first minimum of the radial distribution function between water oxygen and non-hydrogen protein atoms.

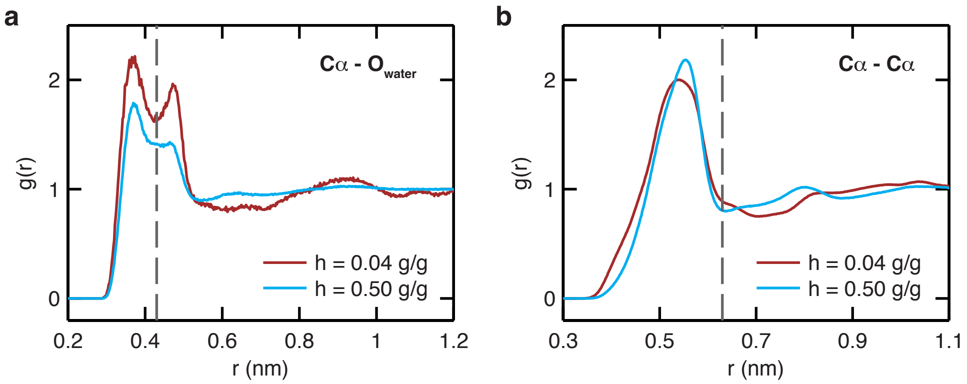

Figure S3. (a) α-carbon-water-oxygen and (b) α-carbon-α-carbon radial distribution functions (g(r)) of amino acid mixture at two hydration levels of 0.04 and 0.50 g/g. Gray dashed lines at 0.43 nm and 0.63 nm indicate the cut-off distance used to determine the first hydration and α-carbon coordination shells of α-carbons, respectively.
